# Identifying a Novel DPYD Polymorphism Associated with Severe Toxicity to 5-FU Chemotherapy in a Saudi Patient

**DOI:** 10.1155/2019/5150725

**Published:** 2019-08-21

**Authors:** Nedal Bukhari, Faisal Azam, Mohammed Alfawaz, Mohammed Zahrani

**Affiliations:** ^1^Department of Medical Oncology, King Fahad Specialist Hospital in Dammam, Saudi Arabia; ^2^Department of Internal Medicine, King Fahad University Hospital, Imam Abdulrahman Bin Faisal University, Dammam, Saudi Arabia; ^3^Department of Internal Medicine, University of Jeddah, Jeddah, Saudi Arabia; ^4^Department of Internal Medicine, King Abdulaziz University, Jeddah, Saudi Arabia

## Abstract

Dihydropyrimidine dehydrogenase (DPD) is the major enzyme in the catabolism of 5-Fluorouracil (5-FU) and its prodrug capecitabine. We report a 65-year-old female with rectal adenocarcinoma who experienced severe toxicities secondary to standard dose 5-FU based chemotherapy. She was found to be heterozygous for rs371313778, c.2434G>A. This finding prompted restarting 5-FU at 50% dose reduction with further titration in subsequent cycles. We herein report the first case of rs371313778, c.2434G>A (p.Val812lle)* DPYD* polymorphism leading to severe 5-FU toxicities. The patient eventually completed a 6-month course of adjuvant treatment with modification of 5-FU dose.

## 1. Introduction

Fluoropyrimidines (FLPN), which include 5-FU, its prodrug capecitabine, and tegafur, are chemotherapeutic agents widely used in the management of different types of cancers. Treatment with FLPN is generally well tolerated; a proportion of patients seldom experience grade 3 (G III) and grade 4 (G IV) toxicities after being exposed to them [1–3].

Complete or intermediate deficiency of DPD can result in severe, life-threatening side effects including severe mucositis, diarrhea, and pancytopenia. Genetic polymorphism in its encoding gene* DPYD* has an established impact on enzyme level and toxicity risk [1, 4].

To our knowledge this is the first ever case reported of this novel mutation in the* DPYD* gene associated with severe toxicity to 5-FU-based chemotherapy reported.

## 2. Case Presentation

A 65-year-old female with an initial diagnosis of locally advanced distal rectal adenocarcinoma staged at T2N2, underwent neoadjuvant long course concurrent chemoradiation with capecitabine at 625 mg/m2 twice daily on radiation days. She required an abdominoperineal resection (APR) and end colostomy. She was found to have a left lower lobe nodule highly suggestive of metastatic disease for which she had metastasectomy 6 weeks following her APR. Resected lesion was consistent with primary rectal adenocarcinoma.

The patient received her first cycle of adjuvant Xelox regimen, which consists of capecitabine 1000 mg/m2 for 14 days and oxaliplatin given at 130 mg/m2 on day 1.

A few days after starting treatment, she developed diarrhea that progressed to grade (G) III and did not subside with antidiarrheal medications. Capecitabine was stopped on day 8 of chemotherapy cycle. Diarrhea subsequently resolved in 8 days. GIII fatigue and GII mucositis were also reported as per Common Terminology Criteria for Adverse Events (CTCAE criteria). Laboratory investigations reported white blood cell (WBC) counts of 3.5 X 10 9/L (normal range 4.5-11 X 10^9^/L), neutrophils were 1.58 (normal range 2.5-7), platelets were 137 X109/L (normal range 150-450 x 10^9^), and hemoglobin (Hb) was 7.3 g/dL (normal range 12-17).

Based on the toxicities reported, she was switched to modified FOLFOX-6 regimen every 2 weeks. The patient received a bolus of 5-FU, dosed at 400 mg/m2 on day 1 of treatment plus leucovorin, followed by 46 hours of 5-FU infusion given at 2400 mg/m2. Oxaliplatin was delivered at 85mg/m2. Four days after completion of 5-FU infusion, she developed GIII mucositis, GIII diarrhea, and fever for which she required hospital admission. She was clinically dehydrated, septic, and pancytopenic. Her WBC count was 0.42 X10^9^, neutrophils were 0.15, Hb was 7.4 g/dL, and platelets were 73 X10^9^.

She developed alopecia and phlebitis immediately after receiving one cycle of FOLFOX.

During her hospital admission, she received intravenous (IV) piperacillin-tazobactam, IV rehydration. Complete blood counts, kidney function, and electrolytes were monitored on a daily basis. A blood sample was sent to Mayo Clinic for full* DPYD* gene sequencing after obtaining an informed consent.* DPYD* gene sequencing confirmed a heterozygosity for c.2434G>A, rs371313778. The report stated the following:

Variation identified: Het rs371313778

Genomic position: Chr1: 97700416

cDNA change: c.2434G>A (Heterozygous)

Amino acid change: p.Val812lle

Patient symptoms and blood counts improved in 10 days. Subsequent cycle of chemotherapy was restarted after 2 weeks of delay. Dose of 5-FU chemotherapy was reduced by 50% with no change made to oxaliplatin dose. Patient tolerated dose-modified FOLFOX, which was delivered through a peripherally inserted central (PICC) line. 5-FU dose was gradually increased by 5% in subsequent cycle. Sixty percent of the standard dose of 5-FU was tolerated without severe toxicities. G1 peripheral neuropathy was the only oxaliplatin-related side effect noted.

## 3. Discussion

FLPN based chemotherapy is the backbone treatment used in neoadjuvant, adjuvant, and palliative chemotherapy protocols for different types of cancers, including gastrointestinal, breast, and head and neck malignancies. FLPN are generally well tolerated; however one-third of patients may experience GIII and higher toxicities [5].

DPD is the enzyme that inactivates >80 % of FLPN and converts it to 5,6-dihydrofluorouracil. Forty to sixty percent of patients with severe side effects will have partial or complete enzyme deficiency [6, 7]. There is a 10-fold increase in 5-FU half-life in those patients, leading to severe toxicities with the standard dose. Approximately 3–5% of Caucasians have intermediate DPD deficiency and 0.2% have complete DPD deficiency [1, 5].

*DPYD*, the gene encoding DPD, is a large gene with 4,399 nucleotides in 23 coding exons spanning 950 kb on chromosome 1p22 [7–9]. The* DPYD* gene is extremely polymorphic with over a hundred mutations reported so far [10]. A few of those variants that associate with DPD deficiency are clinically validated through genotype-guided dosing adjustment. Polymorphisms such as c.1905+1G>A, rs3918290 (*∗*2A), c.2846A>T, rs67376798 (D949V), I560S c.1679T>G (*∗*13), rs55886062, c.1129–5923C>G, and rs75017182 (HapB3) are most common variants described in Caucasian population [8, 9, 11].

Of these variants,* DPYD ∗*2A and DPYD *∗*13 have the most deleterious impact on DPD activity, whereas D949V and c.1129–5923C>G result in moderately reduced DPD levels [7, 8]. Screening for those* DPYD* polymorphisms prior to starting 5-FU-based chemotherapy, with genotype-based dosing, has been validated by the clinical pharmacogenetics implementation consortium (CPIC) [8, 9]. However, to date, routine screening for* DPYD* polymorphisms is only performed in few centers. Moreover, the peripheral blood enzyme assay has not been sufficiently effective in predicting 5-FU clearance or severe toxicity to warrant routine use [8, 9, 11].

The genotype sequencing of our patient was heterozygous for* DPYD* rs371313778, c.2434G>A (p.Val812lle). This variant has been previously reported to have normal activity in vitro with uncertain significance. It has been observed at low frequencies in individuals of various ethnic groups (0.01-0.07 minor allele frequency) [12].

A thorough literature search was conducted and, to the best of our knowledge, FLPN toxicity related to* DPYD* rs371313778, c.2434G>A (p.Val812lle) has never been reported. Genotype-based dosing was applied to the treatment course of our patient in subsequent cycles as recommended for patients with polymorphisms associated with DPD deficiency.

## 4. Conclusion

This case is the first to identify the association of c.2434G>A, rs371313778* DPYD* variant with severe FLPN toxic effects, suggesting that it has utility as a predictive genetic marker. This case report also suggests the benefit from genotype-guided dosing of FLPN in patients carrying this genetic polymorphism.
